# Estimating physical activity and sedentary behaviour in a free-living environment: A comparative study between Fitbit Charge 2 and Actigraph GT3X

**DOI:** 10.1371/journal.pone.0234426

**Published:** 2020-06-11

**Authors:** Marie-Louise K. Mikkelsen, Gabriele Berg-Beckhoff, Peder Frederiksen, Graham Horgan, Ruairi O’Driscoll, António L. Palmeira, Sarah E. Scott, James Stubbs, Berit L. Heitmann, Sofus C. Larsen

**Affiliations:** 1 Research Unit for Dietary Studies, The Parker Institute, Bispebjerg and Frederiksberg Hospital, The Capital Region, København, Denmark; 2 Department of Public Health, University of Southern Denmark, Esbjerg, Denmark; 3 Biomathematics & Statistics Scotland (James Hutton Institute), Aberdeen, Scotland, United Kingdom; 4 School of Psychology, Faculty of Medicine and Health, University of Leeds, Leeds, England, United Kingdom; 5 Centro Interdisciplinar para o Estudo da Performance Humana, Faculdade de Motricidade Humana, Universidade de Lisboa, Lisbon, Portugal; 6 The National Institute of Public Health, University of Southern Denmark, Copenhagen, Denmark; 7 The Boden Institute of Obesity, Nutrition, Exercise & Eating Disorders, The University of Sydney, Sydney, Australia; 8 Department of Public Health, Section for General Practice, University of Copenhagen, Copenhagen, Denmark; Linneaus University, SWEDEN

## Abstract

**Background:**

Activity trackers such as the Fitbit Charge 2 enable users and researchers to monitor physical activity in daily life, which could be beneficial for changing behaviour. However, the accuracy of the Fitbit Charge 2 in a free-living environment is largely unknown.

**Objective:**

To investigate the agreement between Fitbit Charge 2 and ActiGraph GT3X for the estimation of steps, energy expenditure, time in sedentary behaviour, and light and moderate-to-vigorous physical activity under free-living conditions, and further examine to what extent placing the ActiGraph on the wrist as opposed to the hip would affect the findings.

**Methods:**

41 adults (n = 10 males, n = 31 females) were asked to wear a Fitbit Charge 2 device and two ActiGraph GT3X devices (one on the hip and one on the wrist) for seven consecutive days and fill out a log of wear times. Agreement was assessed through Bland-Altman plots combined with multilevel analysis.

**Results:**

The Fitbit measured 1,492 steps/day more than the hip-worn ActiGraph (limits of agreement [LoA] = -2,250; 5,234), while for sedentary time, it measured 25 min/day less (LoA = -137; 87). Both Bland-Altman plots showed fixed bias. For time in light physical activity, the Fitbit measured 59 min/day more (LoA = -52;169). For time in moderate-to-vigorous physical activity, the Fitbit measured 31 min/day less (LoA = -132; 71) and for activity energy expenditure it measured 408 kcal/day more than the hip-worn ActiGraph (LoA = -385; 1,200). For the two latter outputs, the plots indicated proportional bias. Similar or more pronounced discrepancies, mostly in opposite direction, appeared when comparing to the wrist-worn ActiGraph.

**Conclusion:**

Moderate to substantial differences between devices were found for most outputs, which could be due to differences in algorithms. Caution should be taken if replacing one device with another and when comparing results.

## Introduction

To research patterns of physical activity (PA) it is important to have methods that measure activity accurately [1]. Historically, research into the benefits and consequences of PA rely on self-reported data, which are prone to inaccuracy and misclassification [1,2], but the development of accelerometers has provided opportunities for passive, direct monitoring of habitual PA in large populations over a long period [3] as well as reducing the burden on participants. One of the most popular devices for use in research is the ActiGraph, which is often used as the comparison when testing newer devices [4–6]. Commercial accelerometers have also recently gained popularity, owing to the development of low-cost devices and cloud storage capacity, which provides opportunities for surveillance of people in real time [7–9]. Furthermore, they have been found to be useful as intervention tools [10], and the development of wrist-worn accelerometers have the opportunity to increase compliance [11]. In 2017, a total of 115.4 million units were sold, with Fitbit being one of the dominating vendors with a market share of 15.4% [12]. However, the algorithms and technical details are proprietary [13,14] limiting the potential to understand the errors and uncertainty associated with the commercial activity trackers [9].

Despite the numerous studies investigating the validity of Fitbit devices in a laboratory setting [13,15], less research has been done in a free-living environment, although agreement has been shown to vary considerably between laboratory and free-living studies, probably because of the larger variation in activities under free-living conditions [16,17]. Furthermore, most studies have tested the validity of Fitbit devices on normal weight and young adults [8,14,18–24] while few studies have been conducted in older or overweight populations [7,25]. Of the studies testing the validity of wrist-worn Fitbit devices in a free-living setting, the majority have investigated the agreement on step count against a hip-worn research grade accelerometer, where mostly an overestimation by Fitbits was found [7,8,20–22,26–28]. Fewer studies have looked at PA levels with reports of both under- and overestimations [7,8,21–23,28] against a hip-worn research grade device and likewise varying results have been reported on energy expenditure (EE) [14,19,22,28] against research grade devices at different placements. As the technical details of Fitbit devices are unknown, continuous confirmation of the validity of new versions of the activity trackers is required [13].

The aim of this study was to investigate how a popular commercial tracker compare to a widely used research-grade device by examining the level of agreement on steps, sedentary time, time in light activity, time in moderate-to-vigorous PA (MVPA), and EE between the commercial Fitbit Charge 2 (FB) activity tracker and the research-grade activity tracker ActiGraph GT3X (AG) in a free-living environment among middle aged adults with overweight or obesity. The agreement between devices was assessed with pre-defined settings and algorithms from the software, thereby investigating whether the FB could easily replace the AG in studies investigating PA measures.

## Materials and methods

This study was part of the baseline examination in the Danish part of the NoHoW study [29], which is a multi-centre randomised controlled trial focusing on weight loss maintenance in adults after a clinically significant weight loss. As part of the trial, all participants received a FB tracker. Participants were also provided with AG devices for a period of one week. This, however, was an opt-in part of the NoHoW trial and as such, this study is only based on a subset of the NoHoW participants. The study included adults aged ≥18 years with a BMI of ≥25 kg/m^2^ prior to losing ≥5% of their body weight during the last 12 months. Participants were excluded if they were pregnant, breastfeeding, had lost weight due to illness or surgical procedures, been diagnosed with an eating disorder, been diagnosed with any condition that may interfere with increasing mild to moderate PA such as walking, or had recently been diagnosed with Type 1 Diabetes. The NoHoW project is registered in ISRCTN (ISRCTN88405328) and approved by the institutional ethics committees at the participating centres (Capital Region of Denmark: H-16030495; 8-Mar-2017). The NoHoW study has received funding from the European Union’s Horizon 2020 research and innovation programme under grant agreement No. 643309.

### Instruments

The FB (Fitbit Inc, San Francisco, CA, USA) is a rechargeable commercial tri-axial accelerometer-based activity tracker and wrist-worn heart rate monitor. It collects minute-by-minute data from which the proprietary algorithms derive step counts, total EE, active minutes, heart rate, floors climbed, distance and sleep time. These variables are displayed on both the device itself and on the user’s account in the associated app [30].

The AG (ActiGraph, Pensacola, FL, USA), is a research-grade tri-axial accelerometer. It is small, lightweight (27 g), and rechargeable, and typically worn at the waist on an elastic band. In the associated ActiLife software, total steps, activity energy expenditure (AEE), Metabolic Equivalent of Task (MET) scores and PA levels (sedentary, light, moderate, vigorous, very vigorous) can be computed via different algorithms and cut-points [31,32]. These have originally been developed for the AG to be placed on the hip. Though validated AEE algorithms for wrist usage are not available in the ActiLife software, the hip algorithms have been adapted for wrist usage, which can be indicated in the software. However, these scaled algorithms have not yet been validated [33], and discrepancy has been found on EE when comparing measures from the two device locations [34]. Thus, in the primary analyses we compared FB measures to measures from AG placed on the hip. However, as pointed out elsewhere [35,36], comparing movement at different bodily locations will lead to discrepancies due to the different movement patterns of these anatomical locations. Therefore, we also examined whether wearing the AG on the wrist provided different levels of agreement with the FB.

### Procedures

This subset of NoHoW participants attended the baseline visit in March-June 2017. Aim and procedures were explained to participants and both written and oral informed consent was obtained. Standing height was measured to the nearest 0.1 cm, using a stadiometer (Seca 704s; SECA, Germany), and weight to the nearest 0.1 kg using a digital weighing scale (Seca 704s; SECA, Germany). Body mass index (BMI) was calculated as kg/m^2^.

All accelerometer devices were initialised via the proprietary software with participants’ age, gender, height, and weight information. A Fitbit account was made for each participant and the app was downloaded to their personal smartphone or tablet. The FB device was updated to the latest firmware (version 22.53.4) and placed on the preferred wrist of the participant. The Fitbit app was configured to reflect the chosen wrist. Two AG instruments were initialised for each participant using the ActiLife Software (version 5.9.2.0); one for the hip (AG_hip_) and one for the wrist (AG_wrist_). The AG_hip_ was placed on the right side of the body, while AG_wrist_ was placed on the same wrist as the FB device. The devices were initialised to collect data for seven consecutive days with a sampling frequency of 30 Hz and an epoch length of five seconds without the low-frequency extension. Participants were instructed to wear the devices for all seven days, excluding nights, and fill out a wear-time log.

### Data processing

Data from both AG devices were downloaded using ActiLife and wear time validation was performed. Non-wear time was defined as 90 consecutive minutes of zero counts, allowing for up to two-minute interruptions of non-zero counts [37]. A minimum of 10 hours of wear time was necessary for a day to be considered valid and a minimum of three valid days was required to be included in analyses. To be able to compare FB data to both AG_hip_ and AG_wrist_, data was excluded from all devices if either AG_hip_ or AG_wrist_ indicated periods of non-wear. Non-wear time from all devices were also removed according to the log filled out by each participant. To exclude possible instances where one tracker was worn but another not, days were also excluded from the analysis, if less than 1500 steps on a device were accumulated over the entire day. This criterion of <1500 was based on a study by Tudor-Locke et al who compared accelerometers located at different positions [38].

### Physical activity outputs

For all AG outputs, the scoring options already available in the ActiLife software were utilised. The vector magnitude (VM = *√((Axis 1)*^2^*+(Axis 2)*^2^*+(Axis 3)*^2^*))* [31,39] was used and for AG_wrist_, the “Worn on wrist” option in ActiLife was applied. Total step count was calculated through the proprietary software for each device. For PA levels, the cut-points for AG_hip_ were <200 VM counts/min for sedentary, 200–2689 VM counts/min for light and ≥2690 VM counts/min for MVPA [40,41]. For AG_wrist_ the chosen cut-points were <2000 VM counts/min for sedentary, 2000–7999 VM counts/min for light and ≥8000 VM counts/min for MVPA [41]. These were manually applied in ActiLife as no predefined cut-points were available. For FB, the PA levels are scored into 4 categories (*sedentary*, *lightly active*, *fairly active*, *and very active*), and it was assumed that combining *fairly active* and *very active* would correspond a MVPA category [21]. When assessing EE from the AG, the Freedson VM3 Combination algorithm in the ActiLife software was used as this algorithm was the one available in the ActiLife software that uses information from all three axes and is developed for data from an adult population [40,42]. The EE output from FB is provided in total calories, while the output for AG is only the active calories. Thus, the estimated basal metabolic rate calculated by the FB was subtracted from the total EE variable from FB to create an AEE variable comparable to AG.

### Statistical analysis

Agreement between devices was assessed using Bland-Altman plots to calculate absolute bias and 95% limits of agreement (LoA). The Bland-Altman plot was originally developed for data with two sets of measurements on one occasion, and hence the mean difference and LoA are quite simple to calculate [43]. However, that would require the measurements for each person to be grouped into weekly averages and some of the day-to-day variation is lost [44,45]. To keep the daily variation in the data, observations were not aggregated into weekly averages per participant, but each day consisted of a paired observation. As such, observations would be naturally clustered within participants and therefore, to take this clustering into account, a multilevel analysis with a random term for participant ID and no fixed effects was performed to assess mean difference and LoA. Bias was assessed though visual inspections of the plots. All analyses were performed using Stata SE 15.0 (StataCorp LP, College Station, Texas, USA; www.stata.com).

#### Sensitivity analyses

A sensitivity analysis was performed where FB was compared to AG_wrist_ instead of AG_hip_. To compare how the agreement would change, a replicate of the main analysis was conducted using the AG_wrist_ data. Furthermore, most studies comparing activity trackers with a duration of >1 day aggregate data into weekly averages [7,8,23,27,28]. It is likely that this would provide different results as some of the variation disappears [46]. To investigate this, an additional analysis was conducted where the observations for FB and AG_hip_ from the main analysis were grouped into weekly averages and a traditional Bland-Altman plot constructed to examine how much the results change.

## Results

Out of 536 recruited NoHoW participants in Denmark, 143 attended the baseline visit in March-June 2017. Of these, 60 participants agreed to take part in this study. After exclusion on non-compliance to protocol and wear-time criteria, the analysis included 41 participants with a total of 256 valid days and an average of 6.2 days recorded per participant. Participant characteristics can be found in Table 1.

**Table 1:** Participant characteristics.

	All (n = 41)	Men (n = 10)	Women (n = 31)
Age (years)	47.6 (10.4)	48.4 (11.0)	47.4 (10.4)
BMI (kg/m^2^)	29.4 (4.8)	30.4 (6.0)	29.1 (4.4)
Valid days per participant	6.2 (1.1)	6.4 (1.0)	6.2 (1.2)
Daily wear time per participant (hours)	14.1 (1.1)	14.6 (1.1)	13.9 (1.1)

Results presented as mean (SD)

Compared to AG_hip_, FB provided higher mean measures on steps, time in light activity and AEE, and lower mean measures on time in sedentary and MVPA. Compared to AG_wrist_, FB provided higher mean measures on time in sedentary and lower mean measures on steps, time in MVPA, and AEE (Table 2).

**Table 2:** Instrument variability.

	Fitbit			AG_hip_			AG_wrist_		
Included days [n]	256								
	Mean (SD)	Min	Max	Mean (SD)	Min	Max	Mean (SD)	Min	Max
Steps [n/day]	10,209 (4,742)	1,687	25,493	8,753 (4,324)	1,697	23,217	11,659 (3,821)	3,116	22,458
Sedentary [min/day]	551 (122)	234	878	574 (118)	228	900	376 (111)	104	719
Light [min/day]	242 (84)	73	511	183 (64)	67	394	92 (28)	28	277
MVPA [min/day]	59 (53)	0	245	91 (50)	0	268	380 (106)	158	672
AEE [kcal/day]	1,105 (535)	205	3,129	701 (397)	143	2,219	2,660 (1,177)	158	7,261

MVPA, moderate-to-vigorous physical activity; AEE, activity energy expenditure

### Assessing agreement

**Fig 1:**
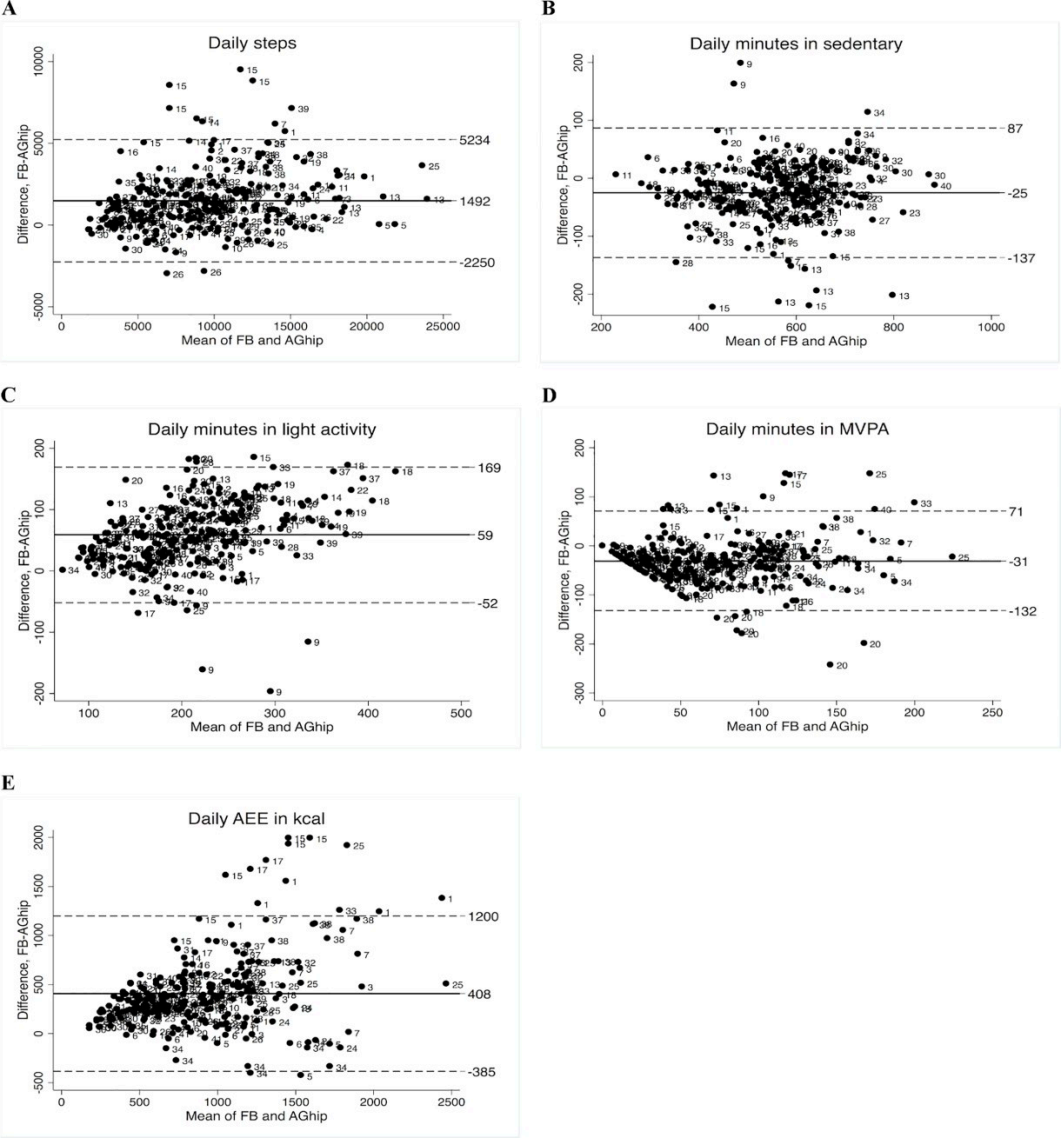
Bland-Altman plots of differences between wrist-worn Fitbit Charge 2 and hip-worn ActiGraph against the mean on: (A) Steps, (B) Time in sedentary, (C) Time in light activity, (D) Time in MVPA, and (E) AEE. The bold lines represent mean of the differences between devices, the dashed lines are the 95% limits of agreement. The numbers in the plot represent each subject. Abbreviation’s: FB, Fitbit Charge 2; AGhip, ActiGraph GT3X worn on the hip; MVPA, moderate-to vigorous physical activity; AEE, active energy expenditure.

Bland-Altman plots with mean difference and LoAs comparing FB to AG_hip_ for each variable are presented in Fig 1. FB measured 1,492 more daily steps than the AG_hip_ (LoA = -2,250; 5,234). The Bland-Altman plot (Plot A) displayed an even spread of observations around the mean difference and thus no proportional bias. The difference in sedentary behaviour between devices was small with a bias of -25 min/day (LoA = -137; 87) (Plot B). For time in light activity, FB measured 59 min/day (LoA = -52; 169) more than AG_hip_. In the Bland-Altman plot (Plot C) the observations form a slight upwards trend, suggesting a small increasing positive bias with higher daily time in light activity. For time spent in MVPA, there was a mean difference of -31 min/day (LoA of -132; 71). Due to the funnel structure of the plot, it can be seen, that as time in MVPA increased, the difference between devices increased (Plot D). Furthermore, an unusual distribution of observations in the Bland-Altman plots for MVPA could be seen. Also, for AEE (Plot E), high discord was found between FB and AG_hip_. FB measured a mean of 408 kcal/day (LoA was -385; 1,200) more than AG_hip_. Furthermore, as the kcal increased the agreement between devices decreased.

### Sensitivity analyses

When comparing FB to AG placed on the wrist instead of the hip a generally higher discord between devices was observed (S1 Fig). FB measured 1,433 (LoA -5,875; 3,010) fewer daily steps than AG_wrist_. As the number of steps increased, so did the difference between devices. For time in sedentary, the mean difference between devices was 173 min/day (LoA = 32; -314). For time in light activity, the difference between devices was 150 min/day (LoA = -23; 323). The slight increasing positive bias seen in the plot when comparing FB and AG_hip_ became much more pronounced when comparing to AG_wrist_. For time spent in MVPA, the mean difference was -320 min/day (LoA = -523; -117) when compared to AG_wrist_. For AEE, the FB measured 1,563 kcal/day (LoA = -3,340; 215) less than AG_wrist_. In the Bland-Altman plot, the observations form a downwards trend, suggesting an increasing negative bias with higher daily AEE.

Analyses of weekly averages for all variables comparing FB to AG_hip_ were also performed and presented as Bland-Altman plots (S2 Fig). The mean differences were 1,500 daily steps (LoA = -1,237; 4237), -25 min/day (LoA = -121; 70) for time in sedentary, 59 min/day (LoA = -28; 145) for time in light activity, -30 min/day in MVPA (LoA = -111; 51), and 408 kcal/day (LoA = -240; 1056) for AEE.

## Discussion

The present study compared several physical activity estimates from a commercial activity tracker, FB, to a research grade accelerometer, AG, worn on the hip and wrist. When compared to the AG_hip_, a moderate error was observed for steps and substantial error was observed for AEE and MVPA. Furthermore, for MVPA and AEE the disagreement between the commercial and the research grade devices seemed to increase as the mean measure increased.

Similar to these findings, most but not all other studies comparing a wrist-worn Fitbit device to AG also found that a Fitbit measured more steps compared to AG. Chu et al. [26] and Alharbi et al. [7] reported that the Fitbit Flex measured 1,300 steps/day and 1,461 steps/day more than AG, respectively. Brewer et al. [23], who utilised a protocol of testing several different Fitbit devices, found that they measured 1,365 steps/day more than AG. Similarly, Farina and Lowry [27] and Hargens et al. [28] reported that the Fitbit Charge measured 2,690 steps/day and 1,695 steps/day more than AG, respectively, while Sushames et al. [22], contrarily reported that Fitbit Flex measured less daily steps by 3,313. It should be noted that in this latter study, activity was only measured for a single day, which may not give the full picture of variety in activities in daily life. Furthermore, all these studies, except for two [7,27] tested validity on young, normal weight adults.

For time in sedentary behaviour, the present results contrast with previous studies, which indicated larger discord. Dominick et al. [8] reported a difference of 26%/day and Reid et al. [21] reported a difference of 1.28 hours/day when comparing Fitbit Flex to AG. The studies by Dominick et al. [8] and Reid et al. [21] also reported results for in time in light activity, where they found differences of -34%/day and -5.12 min/day, respectively, when comparing Fitbit Flex to AG. These are both in the opposite direction of the present results. For MVPA, the current results extend on previous research by Reid et al. [21] who found a mean difference of -57.5 min/day between Fitbit Flex and AG. However, no substantial difference was found by Brewer et al. [23] and Hargens et al. [28] who reported a difference of 0.81 min/day and 5.9 min/day, respectively.

Only two other studies have compared a wrist-worn Fitbit to AG on AEE. Sushames et al. [22] reported that the Fitbit Flex overestimated AEE by 269 kcal/day, while Hargens et al. [28] reported that Fitbit Charge overestimated AEE by 580 kcal/day. Both are similar to the results found in the current study.

Our results revealed that there was lower agreement on all outputs between FB and AG_wrist_ than between FB and AG_hip_. To our knowledge, no previous studies have compared FB to a wrist-worn AG. However, a study by Tudor-Locke et al. [38] found that a waist-worn AG performed better in measuring steps than an AG worn on the wrist when compared to manual counting, and that the wrist-worn AG over-counted steps in a free-living setting when compared to the waist-worn AG. Furthermore, the plots showed systematic bias on all wrist outputs except sedentary time. The major reason for the discrepancies on all outputs is probably that cut-points and algorithms for the AG available in the software have been developed with the purpose of hip placement [36,40,47], and therefore may not be applicable for a wrist-worn AG. This is further evident by the mean time in MVPA of approx. 6 hours per day measured by the AG_wrist_. This is much larger than for the other devices, and in contrast to official recommendations and estimates [48]. The suggestion that comparisons should be done with devices at same bodily locations [35], though sensible, seems to provide more problems than benefits, at least if the comparison device is an AG.

As expected [46], using the weekly averages per participant as one observation instead of keeping each day as separate observations provided roughly the same mean difference for all variables, but narrower LoAs. Furthermore, the observation distribution in the Bland-Altman plots remained, though being less pronounced. Agreement studies for accelerometers often use the weekly averages [7,49,50], which may make the agreement appear falsely high. Depending on the intended use of the given device, and whether one wishes to use estimates of the day-to-day variation, this may be an issue of relevance.

Method agreement studies have been criticised for either using the wrong type of analysis, or not reporting the results of Bland-Altman plot adequately [51]. In addition to using Bland-Altman plots as the analytical method, a strength of the present study is keeping the observations as repeated measures for each subject and using multilevel analysis for calculating the LoA. This has rarely been done in earlier agreement studies [52]. In fact, to our knowledge this is the first study to use this method for comparing a Fitbit device to AG outputs.

Our study is limited by some technical details. Firstly, the cut-points for classifying PA levels on AG varies a lot and choosing the right one becomes quite arbitrary. In fact, Loprinzi et al. [53] reviewed the previous literature and found 12 different MVPA cut-point thresholds ranging from 191 to 3285 counts/min. Depending on the cut-point applied to their own data set, the prevalence of adults meeting the official recommendations ranged as much as from 4.7% to 97.5%. Furthermore, the cut-points chosen for AG_hip_ in this study [40] were calibrated under laboratory settings and in a population with a mean age of 28 years, while cut-points chosen for AG_wrist_ were based on a study population with a mean age of 71.9 years [41]. Thus, the chosen cut-points may not be applicable in the given population, of middle aged overweight or obese adults in a free-living context, and this could potentially explain some of the difference when comparing FB to AG_hip_ and AG_wrist_. Furthermore, the study providing these cut-points was not a validation study aiming at finding the most accurate cut-points for wrist usage, but the study did find that these cut-points correlated best with estimates from hip usage. For the FB, there is not much information on how the PA levels are defined, other than the fact that active minutes are earned for activities ≥ 3 METs and may only be registered when there are more than 10 minutes of continuous activity [54], which is quite different from the AG, which is initialised to collect data in five seconds epochs. It is likely this difference in classifying PA levels in the devices have contributed to the level of mean difference in outputs and it may also be the reason for the distinct distribution of observations in the Bland-Altman plots for MVPA. It is very likely that applying the 10 minutes criteria to the AG as well, would have altered these results.

Secondly, there is no information on the specific algorithm used for calculating EE by the FB device. However, a recent meta-analysis showed that devices with optical heart rate sensors are generally more accurate in estimates of energy expenditure. This may indicate that heart rate is incorporated in to the FB algorithms [55], which is not an available feature in the AG. In addition, as with the cut-points, the algorithm used for the AG was developed in a laboratory setting with a sample population with young and fit people [40]. This may not be as precise for a middle-aged, heavier population in a free-living setting as it has been found that higher BMI and slower speed is associated with decreasing accuracy of accelerometer output [7,13,14,18]. Thus, results for the PA levels and AEE in the present study should be interpreted with caution as it is not possible to determine whether the difference between devices is between the actual devices or disparities in cut-points and algorithms. It should be noted that the algorithms and cut-points applied to AG in this study were chosen from those already provided in the ActiLife software. Using raw data and more advanced algorithms and some validated for wrist usage [56,57], it could be beneficial to investigate, how the FB matches different algorithms and cut-points applied to the AG. However, that was beyond the scope of this paper. Future research should further consider that PA outputs of any device could be affected by firmware updates that change the propriety algorithms and settings [58].

Thirdly, as in other studies [7,21,26], the AG is here used as the device to which the newer FB tracker is evaluated against, yet caution should be taken. The AG is not a true criterion measure on PA outputs in a free living context and varying results have been found when testing the validity of AG against e.g. manual counted steps [34,59–61]. For EE, doubly labeled water is considered the gold standard [62] and for measuring steps, it has been suggested that the StepWatch [63,64] is the most accurate while some have attempted the use of video-recording [65]. Neither the doubly labeled water nor video recordings seem feasible options in many situations, and for the other PA outputs no true gold standard exists. Thus, the use of AG as a comparison method in the present study can, as such, be considered a limitation if one wishes to predict the true accuracy of the test method. However, in the present study the aim was not a test of the true accuracy of PA outputs, but merely an investigation of how different device agree, and if it was possible to replace one with the other.

Even though the study population consisted primarily of middle aged overweight and obese women, the present results may be better representative of the general population with respect to BMI and age compared to many other validation studies that use convenience samples from colleges and universities and consisted of young and fit subjects. It should, however, be noted that this study included people, who had either just lost weight or were in the process of losing weight. This may have influenced their PA levels, and hence made our sample less representative of the general population.

## Conclusions

Moderate to substantial differences were found for steps, light activity, MVPA, and AEE when comparing outputs from the commercial FB device to the research grade hip-worn AG. The results of this study expand on the existing results from previous literature investigating the validity of wrist-worn Fitbit devices. Considering these discrepancies between FB and AG, the FB may not be suitable for clinical research, but be more appropriate for studies utilising the immediate feedback function and be helpful in setting goals and monitoring individual progress. A substantial limitation in the use of Fitbit in research is the lack of knowledge about how these devices work. If using either the ActiGraph or Fitbit device, researchers should consider the bodily location of the device and be aware that algorithms, cut-points and other criteria applied to the devices will highly influence the results, especially if comparing results to other studies. It would also be beneficial for comparing purposes if future studies report information about specific models and firmware versions. Furthermore, as long as the technical details of Fitbit devices are not open to researchers, investigating the validity is difficult as it is not possible to know if the observed differences are results of the device itself or, more likely, differences in algorithms.

## Supporting information

S1 Fig Bland-Altman plots of differences between wrist-worn Fitbit Charge 2 and wrist-worn ActiGraph against the mean on: (A) Steps, (B) Time in sedentary, (C) Time in light activity, (D) Time in MVPA, and (E) AEE. The bold lines represent mean of the differences between devices, the dashed lines are the 95% limits of agreement. The numbers in the plot represent each subject. Abbreviation’s: FB, Fitbit Charge 2; AGhip, ActiGraph GT3X worn on the hip; MVPA, moderate-to vigorous physical activity; AEE, active energy expenditure.
(TIF)

S2 Fig Bland-Altman plots of differences between wrist-worn Fitbit Charge 2 and hip-worn ActiGraph against the mean using weekly averages on: (A) Steps, (B) Time in sedentary, (C) Time in light activity, (D) Time in MVPA, and (E) AEE. The bold lines represent mean of the differences between devices, the dashed lines are the 95% limits of agreement. The numbers in the plot represent each subject. Abbreviation’s: FB, Fitbit Charge 2; AGhip, ActiGraph GT3X worn on the hip; MVPA, moderate-to vigorous physical activity; AEE, active energy expenditure.
(TIF)
