# Supplementary figures and images for: Estimating physical activity and sedentary behaviour in a free-living environment: A comparative study between Fitbit Charge 2 and Actigraph GT3X
Source: PLoS One. 2020 Jun 11;15(6):e0234426. doi: 10.1371/journal.pone.0234426 (PMC7289355; doi:10.1371/journal.pone.0234426)

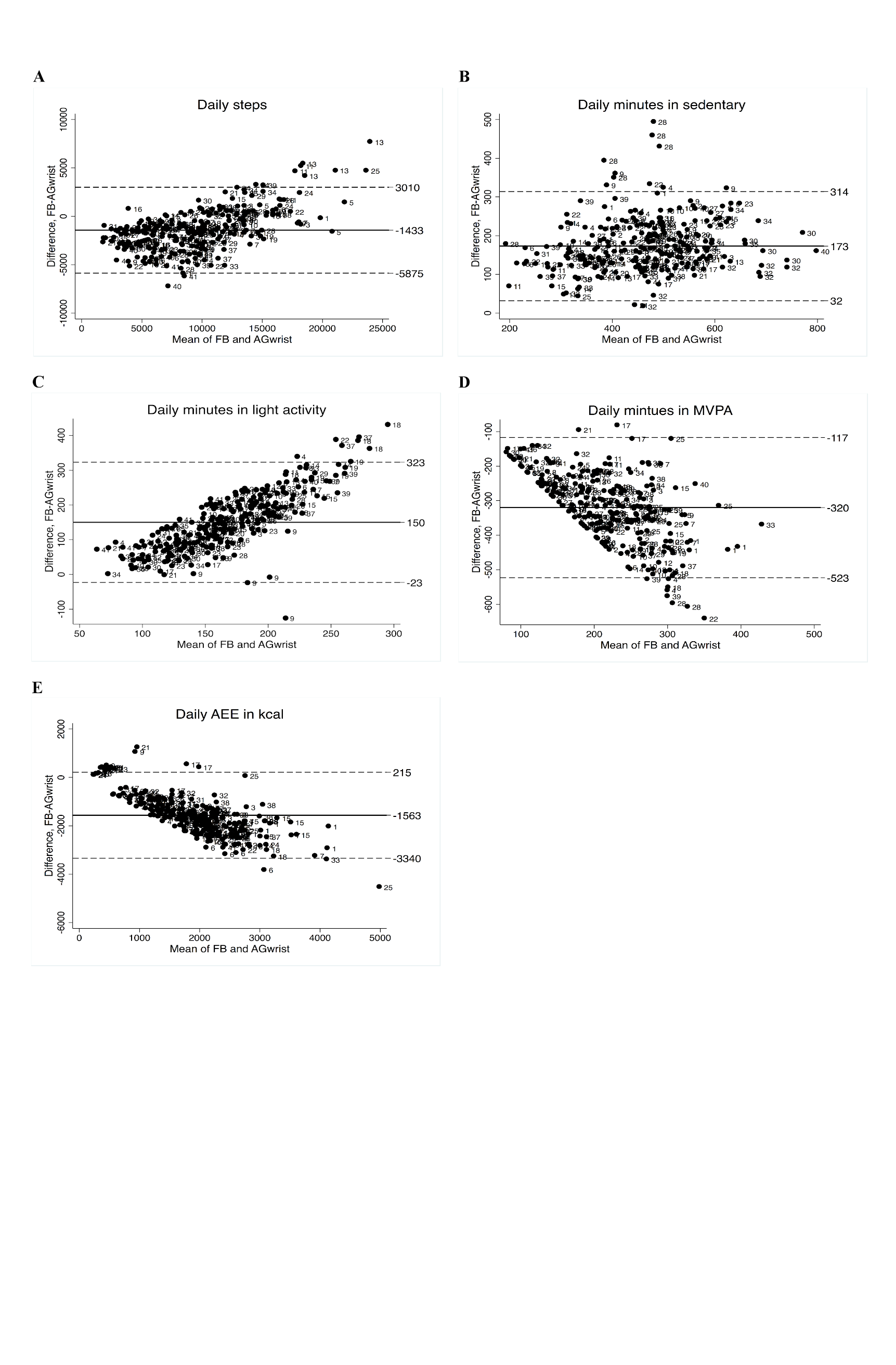

Supplement: S1 Fig — Bland-Altman plots of differences between wrist-worn Fitbit Charge 2 and wrist-worn ActiGraph against the mean on: (A) Steps, (B) Time in sedentary, (C) Time in light activity, (D) Time in MVPA, and (E) AEE. The bold lines represent mean of the differences between devices, the dashed lines are the 95% limits of agreement. The numbers in the plot represent each subject. Abbreviation’s: FB, Fitbit Charge 2; AGhip, ActiGraph GT3X worn on the hip; MVPA, moderate-to vigorous physical activity; AEE, active energy expenditure. (TIF) [file pone.0234426.s001.tif]

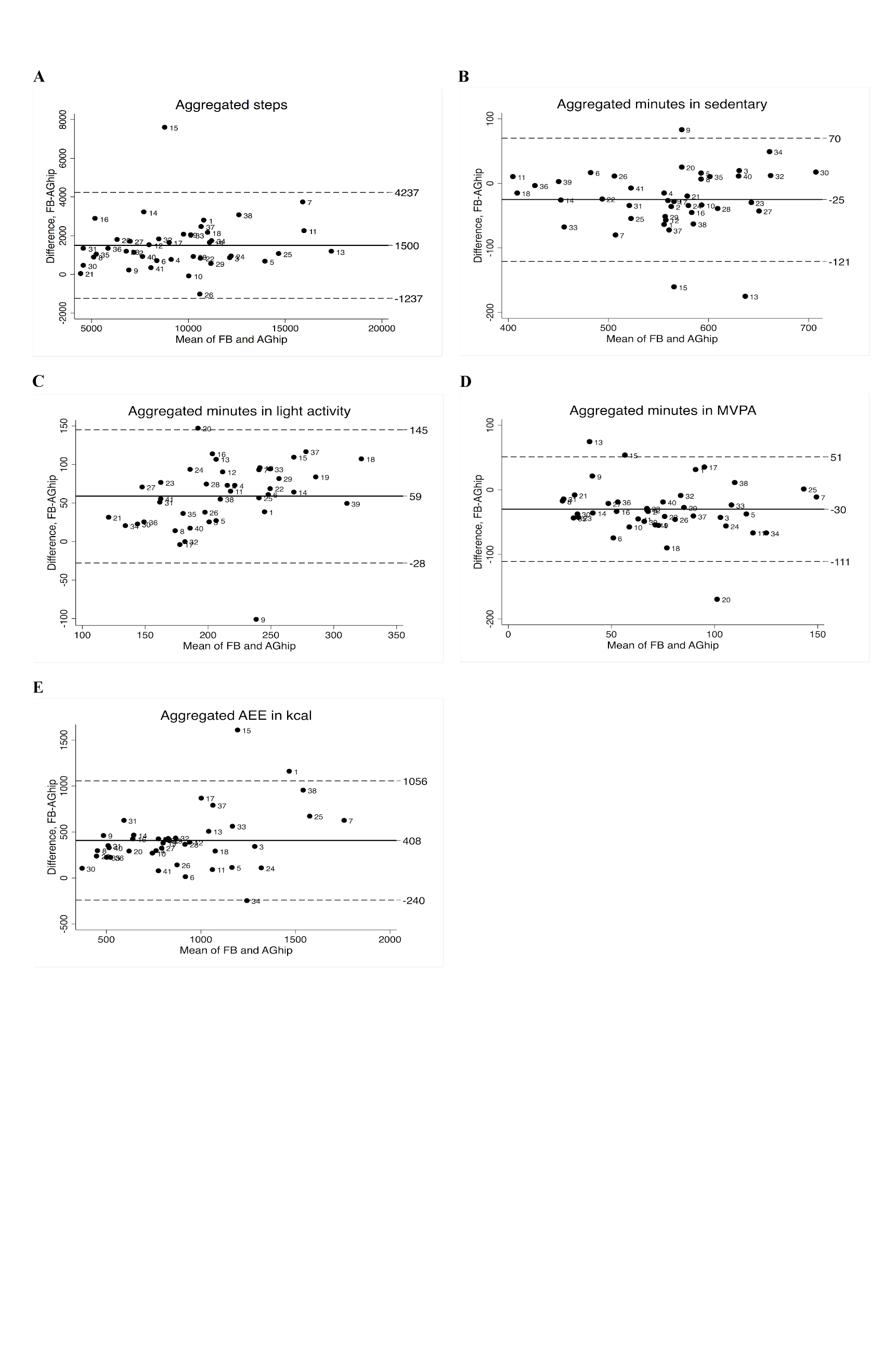

Supplement: S2 Fig — Bland-Altman plots of differences between wrist-worn Fitbit Charge 2 and hip-worn ActiGraph against the mean using weekly averages on: (A) Steps, (B) Time in sedentary, (C) Time in light activity, (D) Time in MVPA, and (E) AEE. The bold lines represent mean of the differences between devices, the dashed lines are the 95% limits of agreement. The numbers in the plot represent each subject. Abbreviation’s: FB, Fitbit Charge 2; AGhip, ActiGraph GT3X worn on the hip; MVPA, moderate-to vigorous physical activity; AEE, active energy expenditure. (TIF) [file pone.0234426.s002.tif]
